# Effect of Lipopolysaccharide on Inflammation and Insulin Action in Human Muscle

**DOI:** 10.1371/journal.pone.0063983

**Published:** 2013-05-21

**Authors:** Hanyu Liang, Sophie E. Hussey, Alicia Sanchez-Avila, Puntip Tantiwong, Nicolas Musi

**Affiliations:** 1 Department of Medicine-Diabetes Division, University of Texas Health Science Center, San Antonio, Texas, United States of America; 2 Geriatric Research Education and Clinical Center, South Texas Veterans Health Care System, Audie L. Murphy Division, San Antonio, Texas, United States of America; 3 Texas Diabetes Institute, San Antonio, Texas, United States of America; John Hopkins University School of Medicine, United States of America

## Abstract

Accumulating evidence from animal studies suggest that chronic elevation of circulating intestinal-generated lipopolysaccharide (LPS) (i.e., metabolic endotoxemia) could play a role in the pathogenesis of insulin resistance. However, the effect of LPS in human muscle is unclear. Moreover, it is unknown whether blockade/down regulation of toll-like receptor (TLR)4 can prevent the effect of LPS on insulin action and glucose metabolism in human muscle cells. In the present study we compared plasma LPS concentration in insulin resistant [obese non-diabetic and obese type 2 diabetic (T2DM)] subjects versus lean individuals. In addition, we employed a primary human skeletal muscle cell culture system to investigate the effect of LPS on glucose metabolism and whether these effects are mediated via TLR4. Obese non-diabetic and T2DM subjects had significantly elevated plasma LPS and LPS binding protein (LBP) concentrations. Plasma LPS (r = −0.46, *P = *0.005) and LBP (r = −0.49, *P = *0.005) concentrations negatively correlated with muscle insulin sensitivity (M). In human myotubes, LPS increased JNK phosphorylation and MCP-1 and IL-6 gene expression. This inflammatory response led to reduced insulin-stimulated IRS-1, Akt and AS160 phosphorylation and impaired glucose transport. Both pharmacologic blockade of TLR4 with TAK-242, and TLR4 gene silencing, suppressed the inflammatory response and insulin resistance caused by LPS in human muscle cells. Taken together, these findings suggest that elevations in plasma LPS concentration found in obese and T2DM subjects could play a role in the pathogenesis of insulin resistance and that antagonists of TLR4 may improve insulin action in these individuals.

## Introduction

Insulin resistance in the skeletal muscle plays a pivotal role in the development of type 2 diabetes mellitus (T2DM). In recent years the gut microbiota has been linked to this pathogenic process. Specifically, lipopolysaccharide (LPS or endotoxin), an outer membrane component of Gram negative bacteria, is considered to be a causative factor for insulin resistance. It has been postulated that high fat containing diets alter gut flora growth and intestinal wall permeability, elevating enterobacterial production and translocation of LPS into the systemic circulation [1]. This phenomenon has been linked to insulin resistance in animal models of obesity and T2DM [1], [2]. Recent studies have also demonstrated elevated plasma LPS concentration in some groups of obese and T2DM subjects [3], [4]. Moreover, a high-fat meal induced an increase in plasma LPS concentration in both healthy and T2DM subjects [5], [6], while LPS administration to healthy subjects rapidly induced insulin resistance [7]. As such, the elevation in LPS concentration could play a key role in low-grade systemic inflammation, which is a central feature of obesity, insulin resistance, and T2DM.

LPS is a key component of Gram negative bacteria cell walls [8]. LPS is taken up by LPS binding-protein (LBP) in the blood and transferred to the TLR4 receptor complex, conformed by dimerized TLR4 and MD-2, to initiate downstream signaling [8]. Soluble CD14 (sCD14) also facilitates LPS activation by transferring monomeric LPS to membrane CD14 (mCD14) complexed with MD-2/TLR4 or by transferring LPS directly to MD-2/TLR4 on cells that do not express mCD14 [9]. LPS binding to TLR4 recruits intracellular adaptor proteins which leads to the activation of pro-inflammatory kinases associated with insulin resistance, including the mitogen-activated protein kinases (MAPK) [c-Jun N-terminal kinase (JNK), p38 and extracellular-signal related kinase (ERK)], and the I kappa B kinase (IKK) complex. Downstream transcription factors such as nuclear factor κB (NFκB) and activator protein-1 (AP-1) increase expression of inflammatory proteins such as tumor necrosis factor-α (TNFα), monocyte chemotactic protein (MCP)-1 and interleukin (IL)-6 [10]. Concordantly, genetic or chemical inhibition of either JNK or IKKβ/NFκB can improve insulin sensitivity [11]. Moreover, disrupted expression of TLR4 protects mice from developing inflammation and insulin resistance in response to chronic changes in dietary fat [12]. Taken together, these findings provide compelling evidence that the insulin signalling cascade is negatively regulated by TLR4.

Skeletal muscle is the principal tissue responsible for insulin-stimulated glucose disposal and is a major site of peripheral insulin resistance [13]. Despite the evidence suggesting that elevated plasma LPS could play a role in the pathogenesis of insulin resistance, it is not known whether LPS impairs insulin action on glucose metabolism in human muscle. The goal of this study was to test whether LPS stimulates TLR4-driven (IKK-NFκB, MAPK) signaling and impairs insulin action in human muscle cells. Also, we tested whether blocking/down-regulating TLR4 using a novel pharmacologic inhibitor (TAK-242) and RNA silencing would prevent the ability of LPS to activate TLR4 and impair insulin action in human muscle cells.

## Materials and Methods

### Ethics Statement

The study was approved by the Institutional Review Board of the University of Texas Health Science Center at San Antonio (UTHSCSA), and all subjects gave written consent.

### Human Subjects

Twelve lean, nine obese non-diabetic, and ten obese T2DM subjects were recruited through local advertisement. All experiments were performed in the Clinical Research Center (CRC) of the Texas Diabetes Institute. All subjects were sedentary and had stable body weight (±1 kg) for at least 6 months. Each subject underwent a medical history, physical examination, screening laboratory tests, and an oral glucose tolerance test (OGTT). Lean and obese non-diabetic subjects had normal glucose tolerance (NGT) based on American Diabetes Association criteria and did not have a family history (first degree relative) of T2DM. Eight T2DM subjects were diet-treated and two subjects took sulfonylureas which were stopped 24 h before all studies to prevent hypoglycemia during fasting. Subjects did not take any other medications known to affect glucose metabolism.

### Hyperinsulinemic-euglycemic Clamp

After an overnight fast, subjects reported to the CRC. A 180-min euglycemic, hyperinsulinemic (160 mU·m^–2^·min^–1^) clamp was performed as described [14]. Insulin-stimulated glucose metabolism (M) was determined as the mean glucose infusion rate during the last 30 min of the clamp.

### Laboratory Analyses

Plasma glucose was measured using an Analox analyzer (Lunenburg, MA) and hemoglobin A1c using a DCA2000 analyzer (Bayer, Tarrytown, NY). Plasma insulin was measured by radioimmunoassay (Diagnostic Products, Los Angeles, CA). Plasma sCD14, TNFα and IL-6 concentrations were measured using ELISA kits from R&D Systems (Minneapolis, MN). Plasma LBP concentration was measured using an ELISA kit from Cell Sciences (Canton, MA). Plasma LPS concentration was determined using a Limulus Amoebocyte Lysate (LAL) assay kit (Lonza, Walkersville, MD) which has a sensitivity range from 0.01 to 1.0 endotoxin unit (EU)/ml. All materials were endotoxin-free.

### Primary Human Skeletal Muscle Cell Cultures

Primary human skeletal muscle cells were generated from satellite cells obtained from young lean healthy subjects, as previously described [15]. Myoblasts were maintained with growth medium [α-minimal essential medium (MEM) supplemented with 10% fetal bovine serum (FBS), penicillin (200 units/ml)/streptomycin (200 mg/ml) and fetuin (0.5 mg/ml)] and were incubated at 37°C in 5% CO_2_. For glucose uptake assays myoblasts were seeded in 12-well culture dishes at a density of 20,000 cells/well. For all other experiments, myoblasts were seeded in 6-well culture dishes at a density of 40,000 cells/well. When myoblasts reached 80–90% confluence, the cells were differentiated into myotubes for 4–6 days in differentiation medium [α-MEM with 2% FBS, penicillin (200 units/ml)/streptomycin (200 mg/ml) and fetuin (0.5 mg/ml)]. All cells utilized were from the third passage.

For Western blotting and the glucose transport assay, cells were serum starved for 24 h and treated with/without 100 ng/ml LPS in serum-free medium. For gene expression analysis, cells were not serum starved and were treated with/without 100 ng/ml LPS in differentiation medium. Prior to LPS exposure, cells were pre-treated with TAK-242 (1 µM; kindly provided by Takeda Pharmaceuticals) or vehicle (DMSO) for 1 h. TAK-242 is a cyclohexene derivative which selectively inhibits TLR4 by binding to Cys747 in the TIR domain of TLR4 [16] and disrupts TLR4 association with TIRAP [17]. TAK-242 remained in the culture medium throughout the experiment. For insulin signaling assays, cells were stimulated with/without insulin (20 nM) for 20 min.

### siRNA Transfection

Gene silencing by siRNA was performed using lipofectamine RNAiMAX (Life technologies, Grant Island, NY) as described [18].

### Measurement of 2-Deoxy-D-[^3^H]Glucose (2-DG) Transport

2-DG transport was measured as described previously [19]. Briefly, myobutes were stimulated with/without 100 nM insulin for 20 min. Cells were washed twice with HEPES-buffered saline (HBS, 140 mmol/L NaCl, 20 mmol/L HEPES, 5 mmol/L KCl, 2.5 mmol/L MgSO_4_, and 1 mmol/L CaCl_2_, pH 7.4). Glucose uptake was assayed by incubation with HBS containing 10 µmol/L 2-DG (1 µCi/mL) for 5 min. Nonspecific tracer binding was determined by quantifying cell-associated radioactivity in the presence of 10 µmol/L cytochalasin B. Medium was aspirated before washing cells 3 times with ice-cold phosphate-buffered saline (PBS). Cells were subsequently lysed in 50 mmol/L NaOH, and radioactivity was quantified using a scintillation counter. Values were normalized to protein concentration, which was determined using the Bradford method.

### Western Blotting

Western blotting was performed as described previously [15]. Band intensity was quantified with ImageQuant TL (GE Healthcare, Piscataway, NJ). The antibodies used were: anti-IκBα antibody (#9242), anti-GAPDH (#2188), anti-phospho-Akt (Ser473) antibody (#9271), anti-Akt antibody (#9272), anti-phospho-(Ser/Thr) Akt Substrate antibody (#9611), anti-AS160 antibody (#2447), anti-phospho-JNK (Thr183/Tyr185) (#4668), and anti-JNK (#9252) from Cell Signaling Technology (Beverly, MA); anti-phospho-IRS-1 (pTyr612) antibody (#I 2658) from Sigma (Saint Louis, MO); anti-IRS-1 antibody (#06-248) from Millipore (Billerica, MA); and anti-TLR4 antibody (#sc-10741) from Santa Cruz Biotechnology (Santa Cruz, CA).

### Gene Expression

MCP-1, IL-6, and TLR4 mRNA expression was measured by quantitative real time PCR using 18S ribosomal RNA (18S rRNA) as an internal control. Quantitative real-time PCR was performed on an ABI Prism 7900HT System (Applied Biosystems, Foster City, CA) using TaqMan One-Step RT-PCR Master Mix Reagents and Assay On-Demand primer/probes (MCP-1: Hs00234140_m1, TLR4: Hs00152939_m1, and 18S: Hs99999901_s1). IL-6 expression was determined using forward primer: 5-GGTACATCCTCGACGGCATCT-3, reverse primer: 5-GTGCCTCTTTGCTGCTTTCAC-3, probe: 5-TGTTACTCTTGTTACATGTCTCCTTTCTCAGGGCT-3. Each sample was run in duplicate. The quantity of mRNA of each gene was determined from the standard curve and then normalized to 18S rRNA.

### Statistical Analysis

Human clinical and laboratory data as well as muscle cell glucose update data are presented as means ± standard error of the mean (SEM). Western blotting and gene expression data are means ± standard deviation (SD). Differences between groups were determined using unpaired Student’s *t*-test or ANOVA, as appropriate, followed by Tukey’s post-hoc analysis. Pearson correlation was utilized to determine correlation values between variables. Statistical significance was assumed at *P*<0.05. Statistical analyses were done using SigmaStat software.

## Results

### Relationship between Plasma LPS and Peripheral Insulin Sensitivity in Human Subjects

Clinical and laboratory characteristics of all subjects are shown in Table 1. On average, lean subjects were nine years younger than the obese and T2DM individuals. However, using analysis of variance we did not find that any of the measured parameters were significantly associated with age. Obese non-diabetic and T2DM subjects had higher body mass index (BMI) compared to lean control subjects. As expected, fasting plasma glucose, fasting plasma insulin, and HbA1c levels were significantly elevated in T2DM subjects (*P*<0.05). Obese and T2DM subjects were more insulin resistant, as evidenced by a significantly reduced M value (*P*<0.05). The average steady state insulin concentrations during the last 30 min of the insulin clamp were 235±20, 219±8, and 264±17 mU/l in lean, obese and T2DM subjects, respectively (*P* = NS). Plasma TNFα and IL-6 concentrations were not different between groups (Table 1).

**Table 1:** Clinical and laboratory characteristics.

Parameters	Lean	Obese	T2DM
n	12	9	10
Age (yr)	39±2	48±3*	48±3*
BMI (kg/m^2^)	25.0±0.6	31.5±1.0*	34.2±1.0*
FPG (mg/dl)	92±3	92±2	151±13*
FPI (pmol/l)	2.7±0.7	4.2±0.9	11.8±1.6*
HbA1C (%)	5.1±0.1	5.0±0.2	7.9±0.6*
M value (mg/kg/min)	10.4±0.7	8.5±0.8*	4.3±0.6*
TNFα (pg/ml)	2.0±0.3	2.2±0.3	2.3±0.5
IL-6 (pg/ml)	2.2±0.3	1.7±0.3	2.5±0.2

All values are mean ± SEM.

* *P*<0.05 vs. Lean. BMI: body mass index; FPG: fasting plasma glucose; FPI: fasting plasma insulin.

**Figure 1:**
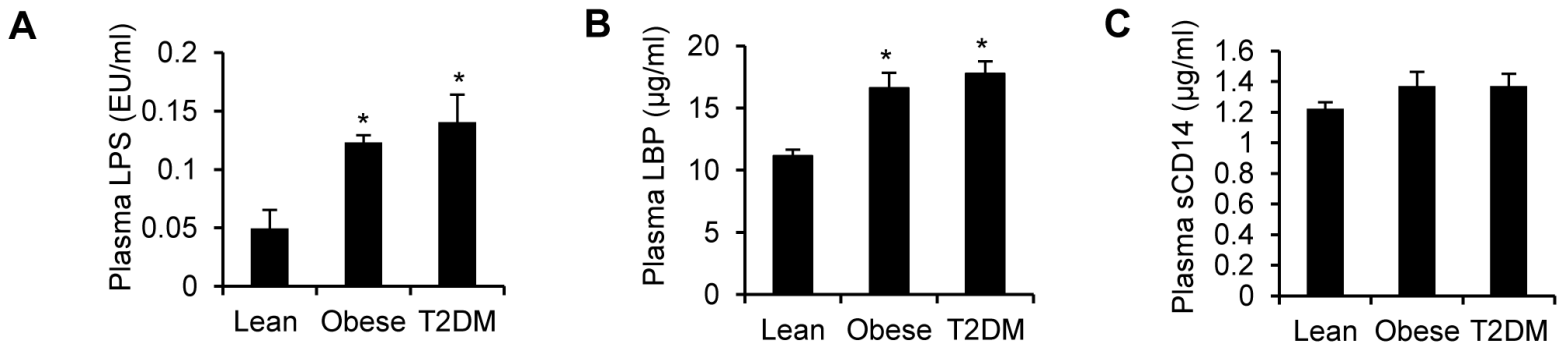
Plasma LPS, LBP and sCD14 levels in insulin resistant subjects. Plasma LPS (A), LBP (B) and sCD14 (C) concentrations were determined as described under ‘materials and methods’. All values are the mean ± SEM of data obtained from 12 lean, 9 obese and 10 T2DM subjects. *P<0.05 compared to lean controls.

Compared to lean subjects, plasma LPS concentration was increased by 2.5- and 2.9-fold in obese and T2DM subjects, respectively (Figure 1A, *P*<0.05). Consistent with the elevated plasma LPS, plasma LBP concentrations were increased in obese and T2DM subjects by 1.5- and 1.6-fold respectively (Figure 1B, *P*<0.05). Accordingly, a positive correlation between plasma LPS and plasma LBP concentrations was observed (r = 0.51, *P = *0.005). Moreover, both plasma LPS (r = −0.46, *P = *0.005) and LBP (r = −0.49, *P = *0.005) concentrations negatively correlated with muscle insulin sensitivity (M) when subjects from the three groups were analyzed together. Within insulin resistant (obese and T2DM) subjects only, LPS (r = −0.29, *P = *0.11) and LBP (r = −0.41, *P = *0.03) also negatively correlated with M. There was a tendency for a small increase in sCD14 in insulin resistant subjects (Figure 1C; *P* = 0.11 and *P* = 0.10 in obese and T2DM subjects, respectively).

### Effect of LPS on Inflammatory Pathways and Insulin Signaling in Human Muscle Cells

**Figure 2:**
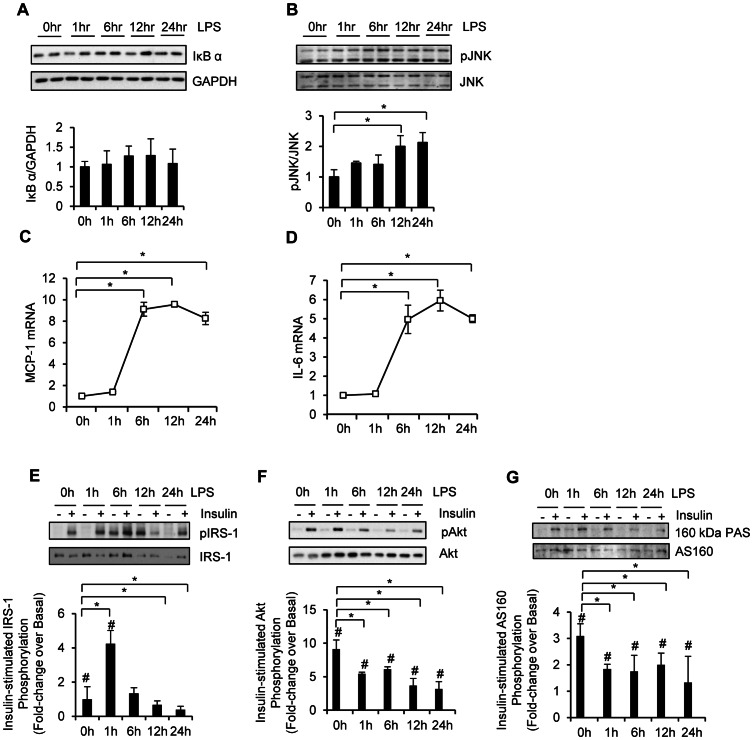
LPS induces inflammation and inhibits insulin signaling in human myotubes in a time-dependent manner. Human myotubes were either left untreated or treated with 100 ng/ml LPS for a varying length of time. The time course of IκBα protein degradation (A), JNK phosphorylation (B), and insulin-stimulated IRS-1-tyr612 (E), Akt (F), and AS160 (G) phosphorylation following LPS treatment was determined by Western blotting. The time course of MCP-1 (C) and IL-6 (D) mRNA expression following LPS treatment was determined by quantitative real time PCR. All values are the mean ± SD of triplicates. *P<0.05; #*P*<0.05 compared to basal.

IκBα protein content was not different after 24 h of LPS treatment (Figure 2A), suggesting that the IKK-NFκB axis was not activated by LPS. In contrast, LPS significantly increased JNK phosphorylation by ∼2-fold within 12 h of stimulation (Figure 2B, *P*<0.05). LPS did not affect p38 phosphorylation (data not shown). In line with the effect on JNK phosphorylation, LPS caused a significant increase in MCP-1 (Figure 2C, *P*<0.05) and IL-6 (Figure 2D, *P*<0.05) gene expression within 6 h of treatment.

At an early time point (1 h) LPS significantly increased insulin-stimulated IRS-1 tyrosine phosphorylation (Figure 2E). At later time points (6, 12, and 24 h) insulin was not able to increase IRS-1 tyrosine phosphorylation, indicative of insulin resistance, although this effect is partially explained by an LPS-induced increase in basal IRS-1 tyrosine phosphorylation (6 and 12 h). At the 24 h time point, the impairment at the level of IRS-1 was fully explained by blunted insulin action, without increases in basal tyrosine phosphorylation. Consistent with the effect on insulin-stimulated IRS-1 phosphorylation, LPS also reduced insulin-stimulated Akt phosphorylation (Figure 2F, *P*<0.05). This inhibition was evident within 1 h and persisted through the incubation. In addition, LPS attenuated insulin-stimulated AS160 phosphorylation throughout the treatment period (Figure 2G, *P*<0.05). Because the effect of LPS on insulin signaling and inflammation reached a peak at 12 h, the following experiments were performed for 12 h.

### Effect of TAK-242 on LPS-induced Inflammation and Insulin Resistance

**Figure 3:**
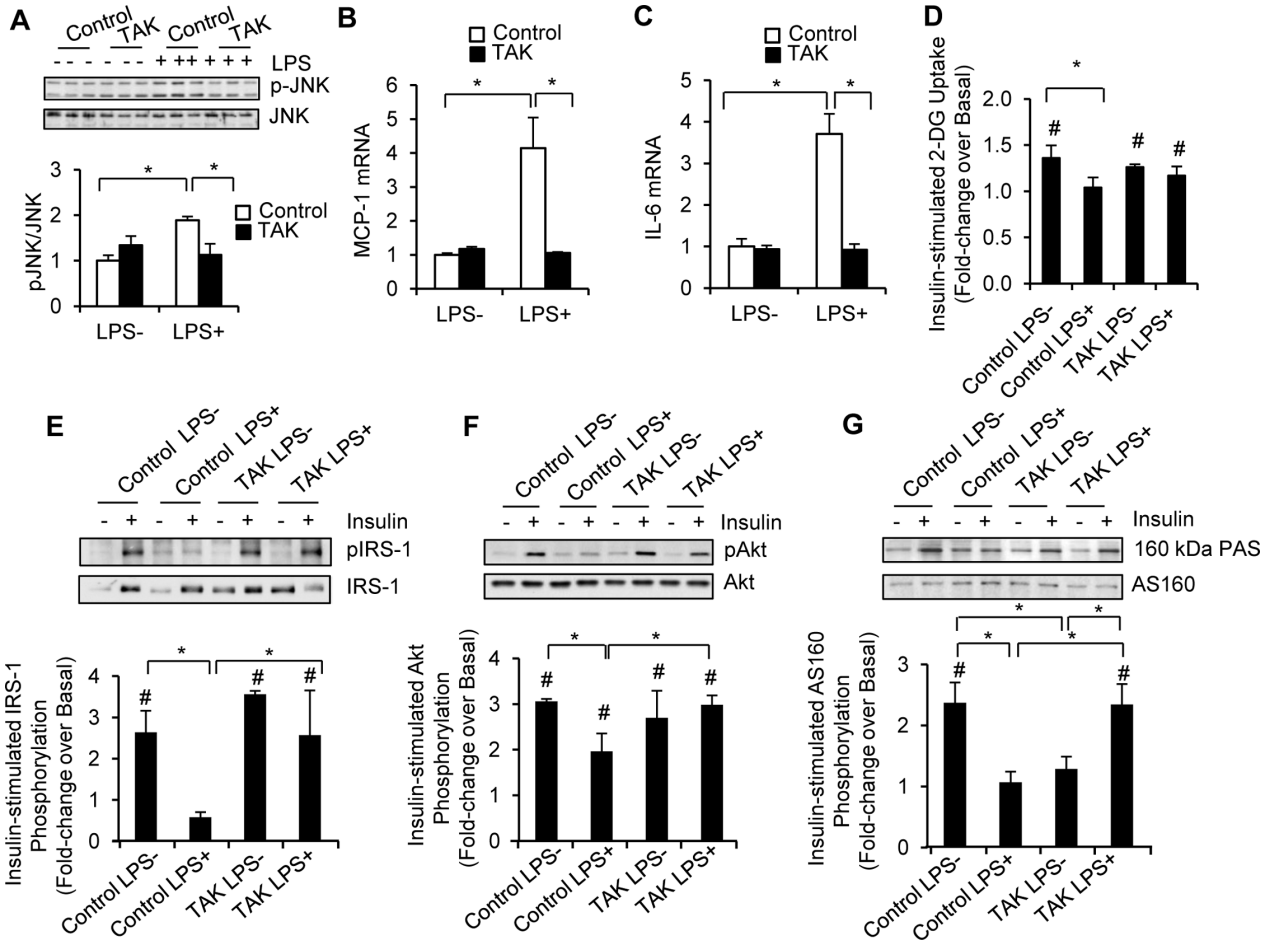
TAK-242 prevents LPS-induced inflammation and insulin resistance in human myotubes. Human myotubes were either left untreated or treated with 100 ng/ml LPS for 12 h. Prior to LPS exposure, cells were pre-treated with 1 µM TAK-242 or vehicle control for 1h. JNK phosphorylation (A), insulin-stimulated IRS-1-tyr612 (E), Akt (F), and AS160 (G) phosphorylation were determined by Western blotting. MCP-1 (B) and IL-6 (C) mRNA expression was determined by real time PCR. Glucose transport (D) was determined by measuring ^3^H-2-DG uptake. The absolute basal glucose transport rates were 43.2±3.2, 46.4±4.1, 46.7±5.6 and 46.5±6.8 pmol/mg,min in cells without LPS, cells with LPS, TAK-242-pretreated cells without LPS, and TAK-242-pretreated cells with LPS, respectively. Results for glucose transport are expressed as the mean ± SEM of data obtained from 5 subjects. All the rest of the values are the mean ± SD of triplicate determinations. Similar experiments were repeated 4 times using cells isolated from different subjects, and representative results are shown. *P<0.05; #*P*<0.05 compared to basal.

To evaluate whether the effects of LPS on inflammation and insulin action can be prevented by inhibiting TLR4 signaling, we pre-incubated the human myotubes with TAK-242. As shown in Figure 3A, TAK-242 fully prevented LPS-induced JNK phosphorylation, as well as the increases in MCP-1 (Figure 3B) and IL-6 (Figure 3C) mRNA expression. The protective effect of TAK-242 on LPS-induced inflammatory responses was accompanied by improvements in insulin-stimulated IRS-1 (Figure 3E), Akt (Figure 3F) and AS160 (Figure 3G) phosphorylation. Notably, TAK-242 alone increased basal AS160 phosphorylation. Therefore, when normalized to basal phosphorylation, insulin-stimulated AS160 phosphorylation was blunted in TAK-242-treated cells. Moreover, LPS inhibited insulin-stimulated glucose transport, while TAK-242 fully restored the ability of the human myotubes to take up glucose in response to insulin (Figure 3D).

### Effect of TLR4 Gene Silencing on LPS-induced Insulin Resistance

**Figure 4:**
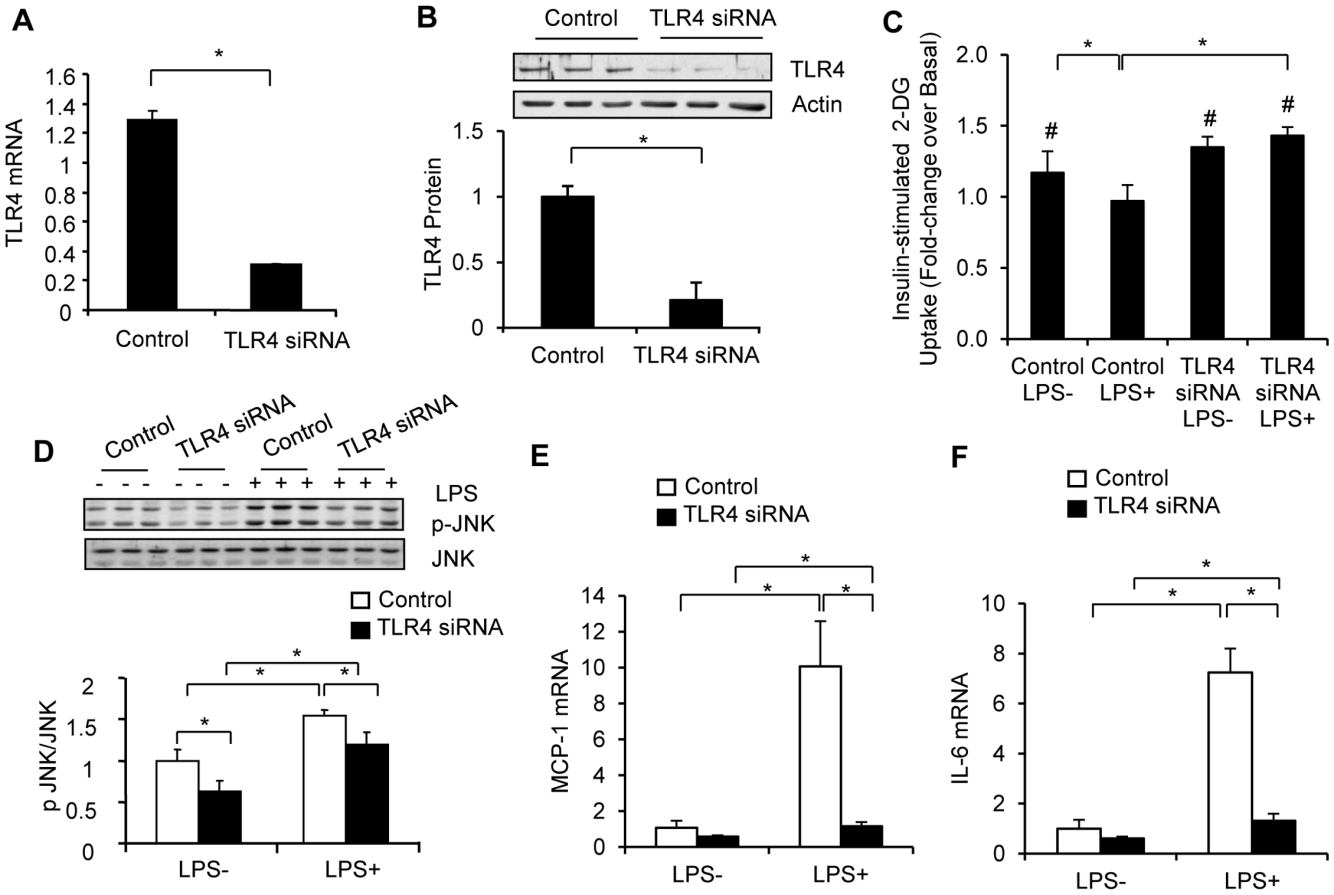
TLR4 silencing protects against LPS-induced inflammation and insulin resistance in human myotubes. Cells were transfected with TLR4 siRNA and negative control siRNA as described under ‘materials and methods’. TLR4 mRNA (A) and protein content (B) were determined by quantitative real time PCR and Western blotting, respectively. Transfected myotubes were either left untreated or treated with 100 ng/ml LPS for 12 h. Glucose uptake (C), JNK phosphorylation (D), and MCP-1 (E) and IL-6 (F) mRNA expression was determined as described above. The absolute basal glucose uptake rates were 30.1±3.2, 35.4±3.9, 30.0±2.1, and 30.7±1.7 pmol/mg,min in the negative control siRNA transfected cells without LPS, negative control siRNA transfected cells with LPS, TLR4 siRNA transfected cells without LPS and TLR4 siRNA transfected cells with LPS, respectively. Results for glucose uptake are expressed as the mean ± SEM of data obtained from 4 subjects. Other values are the mean ± SD of triplicate determinations. Similar experiments were repeated 4 times using cells isolated from different subjects, and representative results are shown. *P<0.05; #*P*<0.05 compared to basal.

We employed gene silencing as an independent, albeit complementary method to examine the role of TLR4 on LPS-induced insulin resistance. TLR4 siRNA decreased TLR4 mRNA (Figure 4A, *P*<0.05) and protein levels (*P*<0.05, Figure 4B) in the human myotubes. TLR4 gene silencing reduced LPS-induced JNK phosphorylation (Figure 4D, *P*<0.05) and the gene expression of MCP-1 (Figure 4E, *P*<0.05) and IL-6 (Figure 4F, *P*<0.05). Notably, TLR4 knock down completely prevented the inhibitory effect of LPS on insulin-stimulated glucose transport (Figure 4C, *P*<0.05).

## Discussion

The present study demonstrates that individuals with obesity and T2DM have increased plasma LPS concentrations. Our findings are consistent with recent studies from different laboratories demonstrating that insulin resistant subject have increased LPS plasma level [4], [6], [20]. In this study we also demonstrate that plasma LPS levels negatively correlate with the M value, which suggests that the inflammatory effect of LPS may be involved in the pathogenesis of muscle insulin resistance.

Our group recently reported that LPS activates JNK, p38, and NFκB in rat-derived (L6) muscle cells [21]. Interestingly, in the present study conducted with human primary myotubes, changes only were observed at the level of JNK. Our findings suggest that selective activation of this Ser/Thr kinase might be the mechanism downstream of TLR4 by which LPS induces insulin resistance in human muscle. Indeed, it is well established that JNK can bind to IRS-1 and cause Ser307 phosphorylation in various cell types [22], leading to decreased activation of phosphoinositide (PI)-3 kinase. In the current study LPS caused a robust increase in MCP-1 and IL-6 gene expression. In cultured human skeletal muscle cells, MCP-1 inhibits Akt phosphorylation and glucose transport [23]. IL-6 also has been reported to impair insulin signaling at the level of IRS-1 through the activation of JNK and suppressor of cytokine signaling (SOCS)-3, and tyrosine dephosphorylation by protein tyrosine phosphatase 1B [24]. Collectively, the activation of JNK and increases in the expression of inflammatory genes (MCP-1, IL-6) caused by LPS in the present study could be responsible for the deleterious effect of LPS on insulin action and glucose metabolism in human cells.

Different mechanisms, not examined during this study, have been implicated in LPS-induced insulin resistance. For example, Pilon et al. showed that LPS treatment in mice induces muscle IRS-1 tyrosine nitration and insulin resistance, an effect that appears to be mediated by inducible nitric oxide synthase (iNOS) [25]. Also, it was reported recently that LPS upregulates endoplasmic reticulum (ER) stress markers in human primary adipocytes [26]. Because ER stress is thought to impair insulin action [27], ER stress also could be a mechanism mediating LPS-induced insulin resistance. In addition, increased biosynthesis of ceramides in response to LPS has been reported [28]. It will be important to determine in future studies whether blocking TLR4 signaling in human myotubes with TAK-242 or siRNA prevents LPS-induced IRS-1 tyrosine nitration, ER stress, and ceramide formation.

Because the skeletal muscle is the main site responsible for insulin-stimulated glucose disposal [13], any process that impairs insulin action in muscle will likely result in altered whole body glucose homeostasis, predisposing individuals to T2DM. Despite the evidence indicating that obese and T2DM subjects have increased plasma LPS concentrations, the direct effects of LPS on insulin action in human muscle is unknown. Here we show that LPS caused a robust inflammatory response in human myotubes, which occurred in concert with impaired insulin signaling and reduced glucose transport. Moreover, we demonstrate, using two different approaches (TAK-242 and siRNA), that blocking TLR4 signaling can prevent the deleterious effect of LPS on insulin action in human muscle. Collectively, these findings suggest that specific pharmacologic interventions that target TLR4 in insulin resistant subjects might be useful to improve glucose metabolism.

LBP binds to the lipid A moiety of LPS with high affinity and facilitates the transfer of LPS to CD14, which in turn interacts with TLR4. LBP is an acute phase protein that is synthesized in the liver upon exposure to LPS [29]. In the present study, also we found that insulin resistant subjects had elevated plasma LBP concentrations, which directly correlated with plasma LPS levels. This association between plasma LPS and LBP levels is likely explained by the ability of LPS to increase LBP gene transcription [29]. How LBP affects LPS action varies, depending upon its concentration. During an acute-phase reaction, such as septic shock, the LBP concentration can rise 10- to 50-fold [29] and inhibit LPS-induced inflammation [30], [31], possibly by transferring LPS to lipoproteins. On the other hand, mild increases in LBP concentration, as seen in insulin resistant subjects, are thought to amplify the cellular response to LPS, in view that LBP increases the bioactivity of LPS by 100-1000 fold [29]. Consequently, a mild elevation in LBP concentration is considered an indication of subclinical endotoxemia in insulin resistant subjects [32], [33].

Frisard et al. examined the effect of LPS on basal (non-insulin-stimulated) substrate metabolism in cultured human skeletal muscle cells [34]. This group reported that a high LPS concentration (500 ng/ml) increased glucose utilization and reduced fatty acid oxidation. Interestingly, a lower LPS concentration (50 pg/ml) also stimulated glucose utilization, but without increasing inflammation gene expression. In the present study we treated human myotubes with 100 ng/ml LPS because this concentration resulted in a modest (∼2-fold) increase in JNK phosphorylation. Also, during preliminary experiments, 50 pg/ml LPS for 12 h did not increase JNK phosphorylation or alter glucose metabolism. Overall, the LPS concentration required to stimulate inflammatory pathways and impair insulin action in muscle cells *in vitro* are considerably higher than the LPS concentration found in serum from insulin resistant subjects *in vivo*. This is explained by the absence of LPS transfer proteins (LBP and sCD14) in the low serum and serum-free culture conditions in the present study. LBP and sCD14 shuttle LPS in a catalytic fashion, accelerating the transfer of LPS by at least 100-fold [35]. When LBP and sCD14 are absent, a much higher LPS concentration is required to exert its cellular effects [36], [37].

The results from this study are consistent with the study by Andreasen et al. who reported that, *in vivo,* LPS administration to human subjects caused an increase in NFκB activity and JNK phosphorylation in muscle [38]. Notably, the response to LPS was more robust in T2DM than NGT subjects. We studied myotubes from young, lean NGT subjects, because we wanted to employ a system that had “normal” glucose metabolism at baseline in order to test the potentially deleterious effect of LPS, and there is evidence indicating that myotubes from T2DM individuals have abnormal glucose metabolism at baseline [39]. Future studies will be aimed at studying whether myotubes derived from T2DM individuals are more susceptible to the negative effect of LPS on insulin sensitivity.

In conclusion, obese and T2DM subjects have elevated LPS/LBP concentrations in the circulation, and LPS directly inhibits insulin signaling and glucose transport in human muscle cells. Pharmacological and genetic inhibition of TLR4 completely protected against LPS-induced inflammation, leading to enhanced insulin action. Our data suggest that metabolic endotoxemia could be involved in the pathogenesis of insulin resistance in obese and T2DM subjects and that targeting TLR4 might be beneficial in these individuals.
